# Arsenic Eaters

**Published:** 1852-05

**Authors:** 


*Arsenic Eaters.*—If the following statements are true they establish some very startling facts. We confess, however, that we have strong doubts whether the human stomach could ever be rendered tolerant of 4 *grains of*

*arsenic*, or 40 *of corrosive sublimate*, per diem. The authority is given by the writer in the London Lancet, who says :—

A trial for murder took place lately in Austria, in which the prisoner, Anna Alexander, was acquitted by the jury, who, in the questions put to the witnesses, in order to ascertain whether the murdered man, Lieutenant Mathew Wurzel, was a poison-eater or not, educed some curious evidence relating to this class of persons. As it is not generally known that eating poison is actually practised in more countries than one, the following account of the custom, given by a physician, Dr. T. von Tschudi, will not be without interest. In some districts of Lower Austria and in Styria, especially in those mountainous parts bordering on Hungary, there prevails the habit of eating arsenic. The peasantry in particular are given to it. They obtain it under the name of *hedri* from the traveling hucksters and gatherers of herbs, who, on their side, get it from the glass-blowers, or purchase it from the cow-doctors, quacks, or mountebanks. The poison-eaters have a two fold aim in their dangerous enjoyment; one to obtain a fresh, healthy appearance, and acquire a certain degree of *embonpoint*. The number of deaths in consequence of the immoderate enjoyment of arsenic, is not inconsiderable, especially among the young. Every priest who has the cure of souls in those districts where the abuse prevails, could tell of such tragedies; and the inquiries made by Dr. Tschudi on the subject have opened out very singular details. The second object the poison-eaters have in view, is to make them, as they express it, "better winded!" that is, to make their respiration easier when ascending the mountains. Whenever they have far to go, and to mount a considerable height, they take a minute morsel of arsenic, and allow it gradually to dissolve. The effect is surprising; and they ascend with ease heights which otherwise they could climb only with effort. The dose of arsenic with which the poison-eaters begin, consists, according to the confession of some of them, of a piece of the size of a lentil, which in weight would be rather less than half a grain. To this quantity, which they take fasting several mornings in the week, they confine themselves for a considerable time; and then gradually, and very carefully, increase the dose according to the effect produced. A strong, hale man, upwards of sixty, takes at present at a dose a piece of about the weight of four grains. For more than forty years he has practised this habit, which he inherited from his father, and which he in his turn will bequeath to his children. It is well to observe, that neither in these nor in other poison-eaters is there the least trace of an arsenic cachexy discernable; that the symptoms of a chronic arsenical poisoning never show themselves in individuals who adapt the dose to their constitution, even although that dose should be considerable. It is not less worthy of remark, however, that when, either from inability to obtain the acid, or from any other cause, the perilous indulgence is stopped, symptoms of illness are sure to appear, which have the closest resemblence to those produced by poisoning from arsenic. These symptoms consist principally in a feeling of general discomfort, attended by a perfect indifference to all surrounding persons and things, great personal anxiety, and various distressing sensations arising from the digestive organs, want of appetite, a constant feeling of the stomach being overloaded at early morning, an unusual degree of salivation, a burning from the pylorus to the throat, a cramp-like movement in the pharynx, pains in the stomach and especially difficulty of breathing. For all these symptoms there is but one remedy—a return to the enjoyment of arsenic. According to inquiries made on the subject, it would seem that the habit of eating poison among the inhabitants of Lower Austria has not grown into a passion, as is the case with the opium-eaters in the East, the chewers of the betel-nut in India and Polynesia, and of the cocoa-tree among the natives of Peru. When once commenced, however, it becomes a necessity. In some districts sublimate of quicksilver is used in the same way. One case in particular is mentioned by Dr. von Tschudi, a case authenticated by the English ambassador at Constantinople, of a great opium-eater at Brussa, who daily consumed the enormous quantity of forty grains of corro sive sublimate with his opium. In the mountainous parts of Peru the doctor met very frequently with eaters of corrosive sublimate; and in Bolivia the practice is still more frequent, where this poison is openly sold in the market to the Indians. In Vienna the use of arsenic is of every-day occurrence among horse-dealers, and especially with the coachmen of the nobility. They either shake it in a pulverized state among the corn, or they tie a bit the size of a pea in a piece of linen, which they fasten to the curb when the horse is harnessed, and the saliva of the animal soon dissolves it. The sleek, round, shining appearance of the carriage-horses, and especially the much admired foaming at the mouth, is the result of this arsenic-feeding. It is a common practice with the farm-servants in the mountainous parts, to strew a pinch of arsenic on the last feed of hay before going up a steep road. This is done for years without the least unfavorable result; but should the horse fall into the hands of another owner who withholds the arsenic, he loses flesh immediately, is no longer lively, and even with the best feeding there is no possibility of restoring him to his former sleek appearance. [The preceding extraordinary statement is abbreviated from a recent number of *Chambers's Edinburgh Journal*, in which it is given as authentic. The use of arsenic in improving the condition of horses is not unfrequent in this country, and its value in the cutaneous diseases of man is established. Such facts, in some degree, confirm these statements; but we entertain grave doubts that an agent of such uniform power as arsenic, could be commenced and habitually continued, in the large doses here mentioned, without producing effects the converse of those described.]

